# *Acrocomia aculeata* Oil-Loaded Nanoemulsion: A Promising Candidate for Cancer and Diabetes Management

**DOI:** 10.3390/ph18081094

**Published:** 2025-07-24

**Authors:** Ariadna Lafourcade Prada, Jesus Rafael Rodríguez Amado, Renata Trentin Perdomo, Giovanna Bicudo Gomes, Danielle Ayr Tavares de Almeida, Leandro Fontoura Cavalheiro, Arquimedes Gasparotto Junior, Serafim Florentino Neto, Marco Antonio Utrera Martines

**Affiliations:** 1 Postgraduate Program in Biotechnology, Faculty of Pharmacy, Food and Nutrition, Universidade Federal do Mato Grosso do Sul, Campo Grande CEP 79070-900, Mato Grosso do Sul, Brazil; lafourcade.ariadna@ufms.br (A.L.P.); renata.trentin@ufms.br (R.T.P.); giovana.bicudo@ufms.br (G.B.G.); 2 Postgraduate Program in Health Sciences, Faculty of Health Sciences, Federal University of Grande Dourados, Dourados CEP 79825-070, Mato Grosso do Sul, Brazil; daniellealmeida@ufgd.eu.br (D.A.T.d.A.); arquimedesjunior@ufgd.edu.br (A.G.J.); 3 Laboratory of Combustibles, Chemistry Institute, Universidade Federal do Mato Grosso do Sul, Campo Grande CEP 79070-900, Mato Grosso do Sul, Brazil; lernfc@gmail.com; 4 Laboratory of Innovation and Development of Drugs and Medicine, Faculty of Pharmaceutical Sciences, Federal University of Amazonas, Manaus CEP 69080-005, Amazonas, Brazil; netosf3@gmail.com; 5 Postgraduate Program in Chemistry, Chemistry Institute, Universiade Federal do Mato Grosso do Sul, Campo Grande CEP 79070-900, Matogrosso do Sul, Brazil; marco.martines@ufms.br

**Keywords:** *Acrocomia aculeata*, bocaiuva, nanoemulsion, diabetes, cytotoxicity, platelet aggregation

## Abstract

**Background:** Diabetes and cancer are two of the most life-threatening disorders affecting individuals of all ages worldwide. This study aimed to develop a novel *Acrocomia aculeata* (bocaiuva) fruit pulp oil-loaded nanoemulsion and evaluate its inhibitory effects on α-glucosidase and pancreatic lipase, as well as its antiglycant activity and cytotoxicity against cancer cells. Additionally, this study assessed the impact of both the oil and the nanoemulsion on blood cells. **Methods:** The pulp oil was extracted by cold pressing. The oil’s physicochemical properties were determined according to the AOAC and the Brazilian Pharmacopeia. The lipid profile was performed by GC-MS. The nanoemulsion was prepared by the phase inversion method using ultrasonic stirring for particle size reduction and for homogenization. Response Surface Methodology was used for optimizing nanoemulsion preparation. Enzyme inhibition tests were conducted using assay kits. Cytotoxicity in cancer cells was evaluated using the Sulforhodamine B assay. **Results:** Comprehensive physicochemical and chemical characterization of bocaiuva oil was performed, identifying oleic acid (71.25%) as the main component. The oil contains 23.04% saturated fatty acids, 73.79% monounsaturated acids, and 3.0% polyunsaturated fatty acids. The nanoemulsion (particle size 173.6 nm; zeta potential −14.10 mV) inhibited α-glucosidase (IC_50_: 43.21 µg/mL) and pancreatic lipase (IC_50_: 41.99 µg/mL), and revealed a potent antiglycation effect (oxidative IC_5_0: 18.36 µg/mL; non-oxidative pathway IC_50_: 16.33 µg/mL). The nanoemulsion demonstrated good cytotoxicity and selectivity against prostate cancer cells (IC_50_: 19.13 µg/mL) and breast cancer cells (IC_50_: 27.22 µg/mL), without inducing hemolysis, platelet aggregation, or anticoagulant effects. **Conclusions:** In this study, a comprehensive physical and chemical characterization of bocaiuva fruit pulp oil was conducted for the first time as a preliminary step toward its future standardization as an active ingredient in cosmetic and pharmaceutical formulations. The resulting nanoemulsion represents a novel alternative for managing diabetes and cancer. Although the nanoemulsion exhibited lower cytotoxicity compared to doxorubicin, it remains promising due to its composition of essential fatty acids, phenols, and carotenoids, which offer multiple health benefits. Further studies are needed to validate its efficacy and safety in clinical applications.

## 1. Introduction

Diabetes and cancer represent two of the most significant global health challenges, contributing to millions of deaths annually and placing an immense burden on healthcare systems worldwide. The relentless progression of these diseases underscores the urgent need for innovative therapeutic strategies that can effectively manage and treat these conditions [1,2]. Traditional medicine has long recognized the potential of natural resources for treating diverse health problems. In this sense, palm tree oils have been widely used by traditional populations for the management of diverse health conditions like malnutrition, obesity, skin lesions, diabetes, and certain types of tumors.

Oils from acurí (*Attalea phalerata Mart. ex Spreng Burret)* and *Cocos nucifera* L. (coconut) have demonstrated promising cytotoxic effects against prostate cancer cells [3]. Lauric acid, a major component of coconut oil, has been shown to inhibit prostate cancer cell proliferation through 5α-reductase inhibition [4,5]. Acurí oil-loaded nanocapsules showed a strong cytotoxic effect against prostate, breast, and renal cancer cells [3]. The pharmacological effect of palm tree oil is associated with its richness in fatty acids, along with other classes of antioxidant phytonutrients like phenolics and carotenoids. By diminishing oxidative damage, palm tree oils could safeguard pancreatic beta cells and bolster insulin function [6]. On the other hand, the high content of polyunsaturated fatty acids in palm tree oils has been associated with a reduced risk of cardiovascular disease and type 2 diabetes mellitus [7]. Nonetheless, despite the great benefits associated with the use of palm tree oils, there are scarce scientific studies validating their ethnopharmacological utilities for developing food and pharmaceutical palm tree oil-based products.

The palm tree *Acrocomia aculeata (Jacq.) Lodd. ex R. Keith* (Arecaceae) is commonly known as bocaiuva and macaúba. This species is widespread in South America, particularly in the west-central region of Brazil [8]. Bocaiuva flour, produced from fresh fruit pulp, serves as the source of bocaiuva oil [9,10]. Bocaiuva oil is traditionally used to treat joint inflammation, colds, diabetes, and skin lesions [10]. These effects are attributed to the fatty acids, phenolics, and carotenoids included in the oil [11,12]. Bocaiuva oil is not toxic and does not affect either platelet aggregation or blood coagulation parameters [13]. Bocaiuva oil in the form of polymeric micelles showed an antiproliferative effect against triple-negative breast cancer cells (MDA-MB-231); however, the pristine oil was inactive [14]. This disparity in activity highlights the critical role of nanoformulations in enhancing the surface area, stability, solubility, and bioavailability of these oils [15].

Nanotechnology is a promising tool for various industries, including pharmaceuticals, cosmetics, and food. The use of simple nanotechnological techniques allows the creation of new materials. Nanotechnology also allows the creation of materials and products with improved surface properties to optimize their functionality [16]. Nanoemulsions can significantly enhance the potency of vegetable oils, enabling lower doses to achieve greater efficacy [16]. This is particularly advantageous for bocaiuva oil, an extractive product with limited annual yield. Therefore, bocaiuva oil-loaded nanoemulsions represent a promising strategy for enhancing pharmacological activity and ensuring sustainable utilization. However, there is a distinct lack of research on the formulation of bocaiuva oil-based products for food, cosmetics, or pharmaceutical applications.

The primary objective of this study is to comprehensively characterize the physicochemical and chemical properties of *Acrocomia aculeata* fruit pulp oil to establish a foundation for standardized quality control in potential pharmaceutical applications. Subsequently, we aim to develop a novel bocaiuva oil-loaded nanoemulsion using a simple, cost-effective method, which will be optimized using a D-optimal Response Surface Methodology. A comprehensive physicochemical characterization of the nanoemulsion will be carried out, its on-shelf stability determined, and its inhibitory effects on pancreatic lipase, α-glucosidase, and protein glycation examined, which are pertinent to diabetes management. Additionally, the antiproliferative properties of the nanoemulsion against prostate and breast cancer cells, the most common cancers in men and women, respectively, will be investigated. Lastly, the hemolytic effects and impact on platelet aggregation of the nanoemulsion will be assessed to ensure its safety and therapeutic potential.

## 2. Result and Discussion

### 2.1. Physicochemical Characterization of Bocaiuva Oil

Bocaiuva oil holds significant economic potential for native populations and could become an important source of income if used as an active ingredient in cosmetics, nutraceuticals, and pharmaceutical products. Bocaiuva oil is a yellowish, translucent, and slightly viscous liquid with a ripe fruit aroma. The color, appearance, and viscosity of this oil change based on the fruit maturity, extraction method, region, soil composition, sunlight exposure, and rainfall. The organoleptic characteristics are consistent with those reported by other authors [9,17].

The physicochemical characterization of bocaiuva oil is crucial for standardizing parameters for assessing its quality and stability. Bocaiuva oil has a relative density of 0.900 (Table 1), akin to that reported by Lescano and coworkers (0.916) [9]. Although density is a crucial characteristic for vegetal oils and plant extract characterization, it has not been consistently reported for bocaiuva oil.

The iodine index indicates the unsaturation level of fatty acids in an oil, reflecting the count of double bonds within the fatty acid chains. Higher iodine indices suggest a high amount of unsaturation [18,19]. The bocaiuva oil showed an iodine index of 74.50 g of I_2_/100 g of oil, which contrasts with the 189.25 g of I_2_/100 g reported by Lescano and collaborators for n-hexane-extracted oil and 182.37 g of I_2_/100 g for pressed oil [9]. Nonetheless, our findings are consistent with the iodine index of 72.01 g of I_2_/100 g of oil reported by Costa and collaborators [20]. The iodine value is a crucial parameter as it helps the assessment of oil oxidation processes over time, the oil’s main stability issue.

In a general way, variations in the iodine index of vegetable oils from the same species can be attributed to several factors. Genetic variability among individual plants leads to differences in fatty acid composition. Environmental conditions, such as soil type, climate, and rainfall, significantly impact the oil’s degree of unsaturation. Agricultural practices, including fertilization, irrigation, and pest control methods, also play a key role. The maturity of fruits at harvest time affects the iodine index, as does the method of oil extraction and processing, with cold-pressed oils often retaining more unsaturated fatty acids. Storage conditions before extraction, including exposure to heat, light, and oxygen, can further influence the oil’s fatty acid profile [18].

The refractive index is a distinctive characteristic of each vegetal oil. This index correlates with the oil’s density and molecular composition and provides insights into the oil’s purity, density, and molecular interactions [16,18]. The refractive index can be used to monitor the quality and consistency of vegetable oils, helping to detect adulteration or degradation of the oil, ensuring the desired physicochemical properties are maintained throughout the extraction process [18]. The refractive index of bocaiuva oil measured at 30 °C was 1.456 ± 0.001, aligning closely with the value reported by Lescano and coworkers, which was 1.450 [9]. The value obtained for bocaiuva oil is probably because oleic acid is the main unsaturated fatty acid present in it, which has a refractive index of 1.459 [18]. In nanoemulsions, the refractive index plays a significant role in determining the stability and optical properties of the formulation. A higher refractive index (usually between 1.46 and 1.49) improves the ability to form smaller droplet sizes, leading to more stable and transparent nanoemulsions [19].

The peroxide index is a critical measure for assessing the oxidative state of an oil. Values exceeding 10 mEq/kg suggest oxidative spoilage [16]. Bocaiuva oil exhibited a peroxide index of 4.50 ± 0.40 mEq/kg, which is significantly lower than 9.25 mEq/kg reported by Costa et al. [17]. This occurs because the oil reported by Costa and coworkers was obtained by extraction with n-hexane, which limits the content of phenolics and carotenes, compounds that prevent peroxide formation in the oil [16].

The saponification value is a metric for the quantity of free fatty acids per gram of oil. It is defined as the mass of potassium hydroxide (in milligrams) needed to neutralize these acids [16,17]. The saponification value of bocaiuva oil was 133.00 mg KOH/g, which is significantly lower than the 255.42 mg KOH/g reported by Nunes and collaborators for kernel oil [12]. This discrepancy is due to the kernel’s distinct fatty acid composition. Despite the various popular uses of bocaiuva oil, including soap manufacturing, there are no reports on the saponification value of this oil. The acidity value of bocaiuva oil was 0.92%, which aligns with the findings of Lescano and collaborators [9].

Bocaiuva oil contains 12.60 mg/g of phenolics and 0.27 mg/g of carotenoids. Carotenoids are responsible for the yellowish color of the bocaiuva oil. Both metabolites are antioxidants and contribute to bocaiuva oil stability. The fatty acid profile obtained by GC/MS (Table 2) shows that bocaiuva oil contains 23.04% saturated fatty acids, 73.79% monounsaturated acids, and 3.0% polyunsaturated fatty acids. Oleic acid was the bocaiuva oil’s main component, representing 71.25 ± 2.21%. This profile agrees with the composition reported by Lescano and coworkers [9] and Costa et al. [17].

The discrepancies in the reported physicochemical properties of bocaiuva oil underscore the necessity for standardized procedures for extraction and characterization. Factors such as cultivation region, seasonal variations, fruit maturity, extraction methods, soil composition, sunlight exposure, and rainfall can affect the bocaiuva oil physicochemical properties. Therefore, any large-scale production of bocaiuva oil intended for industrial applications must undergo standardization in collection, processing, extraction, and storage. This work is the first tentative step toward establishing a complete physical and chemical characterization of bocaiuva fruit pulp oil, as a first step for its future standardization as a row material for industry. However, any large-scale production of bocaiuva oil will need standardization in the collecting, extraction, and storage procedures. This is crucial for promoting the sustainable use of bocaiuva oil and regional development.

### 2.2. Required Hydrophilic–Lipophilic Balance (HLBr) of the Bocaiuva Oil

**Figure 1:**
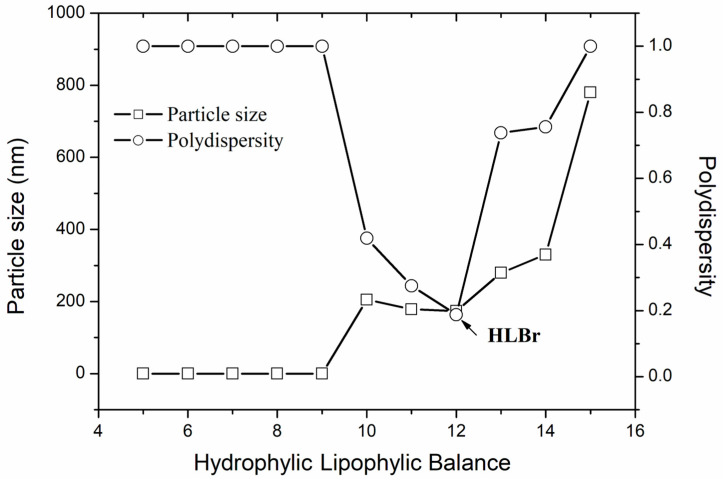
Particle size and polydispersity index of the bocaiuva oil loaded nanoemulsion vs. Hydrophilic–Lipophilic Balance (HLB) of the surfactant mixture (Tween 80/Span 80) used for determining the required HLB of the bocaiuva oil.

To develop a stable and optimized nanoemulsion, it is crucial to determine the required Hydrophilic–Lipophilic Balance (HLBr) of the oily phase (bocaiuva oil). For this, mixtures of Span 80 and Tween 80 were used to reach the desired HLB (from 5 to 15). HLBr suggests a surfactant mixture with a higher probability of producing the most stable and homogenized nanoemulsion [21]. Nanoemulsion number 11 (HLB 11, Figure 1) exhibited a particle size of 185 nm, while nanoemulsion number 12 (HLB 12) displayed a particle size of 190.36 nm. However, nanoemulsion number 11 showed phase separation after 72 h. Contrarily, after 72 h, nanoemulsion number 12 kept the same particle size and the same whitish and turbid appearance, which are signs of homogeneity and stability. The HLBr of bocaiuva oil was set at 12, and this nanoemulsion was chosen for the optimization.

### 2.3. Optimization Process

**Figure 2:**
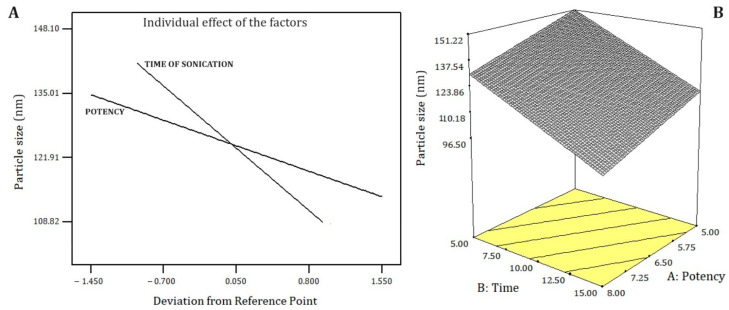
Effect of the sonication time and potency on the particle size of the bocaiuva oil-loaded nanoemulsion 24 h after the preparation. (**A**) Individual linear effects; (**B**) represents both effects combined.

In Table 3 and Figure 2, the results of the nanoemulsion process optimization are presented. In the process optimization, the size and polydispersity index of each nanoemulsion were measured 24 and 72 h after the preparation. To construct optimized graphs, measures obtained at 24 h were used. Nanoemulsions 2, 3, 7, 9, 10, and 15 were discarded because they presented phase separation after 72 h (Table 3). Nanoemulsion 8 increased in size and polydispersity index after 72 h, indicating instability. In contrast, after 72 h, nanoemulsion 14 (sonicated for 15 min at 8 watts) kept a small particle size (139.40 ± 1.75 nm) and its polydispersity index reduced to 0.207, which suggests nanoemulsion stabilization.

The polydispersity index was not affected by the factors (ultrasonic potency and sonication time). Contrarily, the smallest particle size was achieved at the highest levels of both factors (15 min and 8 watts, Figure 2). Particle size fits a linear model (F-test 91.92, *p* < 0.0001), with a strong agreement between Adjusted R^2^ (0.9285) and Predicted R^2^ (0.8838), which is indicative of the model’s robust predictive capacity [22]. A theoretical model with good predictability facilitates the upscale production of the nanoemulsion. The best-fitted model was selected considering the smallest PRESS value of the proposed models and the least difference between Adjusted R^2^ and Predicted R^2^. The factors to be included in the theoretical model were selected based on their significance (*p*-value < 0.05). Equation (1) describes the fitted model as actual factors.

Particle size (nm) = 202.87 − 6.94 × Sonication Potency − 3.40 × Sonication Time(1)

**Figure 3:**
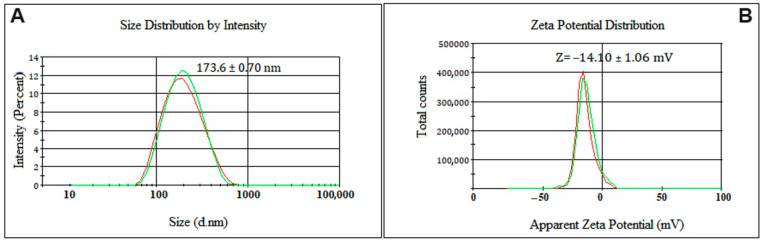
Particle size distribution (**A**) and ζ-potential (**B**) of the optimized nanoemulsion prepared with 5 percent of surfactant with HLB = 12 (28 parts of Span 80 and 0.72 parts of Tween 80), 5 percent of bocaiuva oil, and 90 percent of Milli-Q water. The optimized parameters were sonication potency 8 watts and sonication time 15 min.

The optimized nanoemulsion was composed of 5% surfactant with HLB 12 (28% Span 80 and 72% Tween 80), 5% Bocaiuva oil, and 90% Milli-Q water. The nanoemulsion was produced using a sonication time of 15 min at a power setting of 8 watts. This formulation was replicated three times to ensure its characterization, stability study, and subsequent preclinical pharmacotoxicological evaluation. The same processes were used for a blank nanoemulsion (used as the control in biological assays). The nanoemulsion had a pH of 5.14, making it suitable for pharmaceutical formulations due to its biocompatibility. The particle size of the nanoemulsion was 173.60 nm, with a polydispersity index of 0.199 (Figure 3A) and a ζ-potential of −14.10 mV (Figure 3B). The monomodal size distribution with a close base suggests a high homogeneity in size.

**Figure 4:**
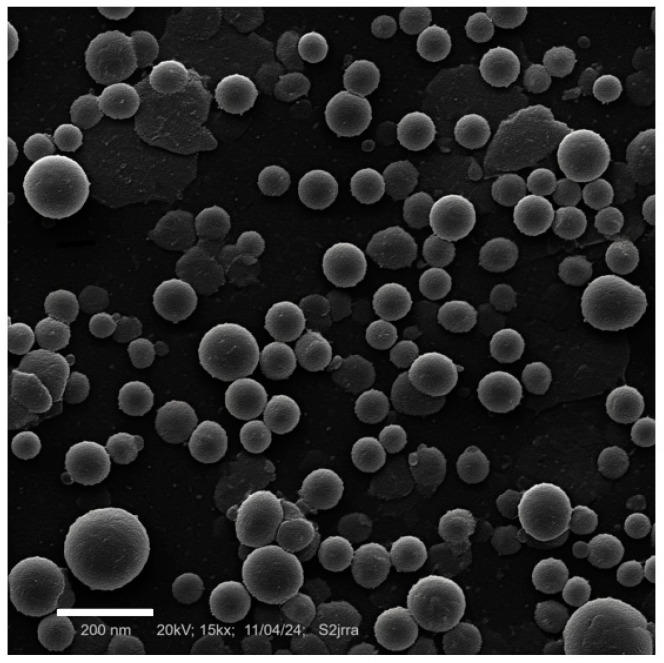
Nanoemulsion particle size and shape obtained by Scanning Electronic Microscopy.

A microphotograph obtained by SEM (Figure 4) shows rounded nanoparticles, with a rough and irregular surface, characteristic of non-ionic nanomicelles. The mean particle size obtained by SEM was 154.25 ± 2.25 nm. The size measured by TEM was slightly smaller than that obtained by DLS (173.60 ± 0.70 nm). This fact occurs because DLS measures the size in a liquid medium, in the presence of dispersants and/or surfactants. The surfactants produce a slight enlargement of size, which can induce errors in DLS particle sizing measurements and shift its results to higher values [23].

### 2.4. Stability Evaluation

The integral stability of nanoemulsions is essential due to the inherent thermodynamic instability of these systems [21]. Assessing the effect of temperature on the physicochemical properties of nanoemulsions is critical to ensuring stability, efficacy, and safety in therapeutic applications. Thermal stability influences key parameters such as solubility, bioavailability, and the controlled release profile of active pharmaceutical ingredients. Furthermore, temperature fluctuations can directly affect a nanoemulsion’s functionality and its interaction with biological systems. Consequently, gaining insight into the behavior of nanoemulsions across a wide temperature range (e.g., 10–80 °C) is crucial for creating therapeutic formulations that retain their desirable properties under different storage and usage conditions.

Throughout this study, the nanoemulsion maintained its appearance, color, and smell without any phase separation or other observable physical changes, indicating stability. The amount of bocaiuva oil, measured by GC, showed no statistically significant differences over time at *p* = 0.05 (F-test 0.85, *p*-value 0.5015). The bocaiuva oil content consistently remained above 98.45% (±2.48%), suggesting its stability over time. This consistency is crucial for preserving the nanoemulsion’s effect and ensuring precise dosage [24].

**Figure 5:**
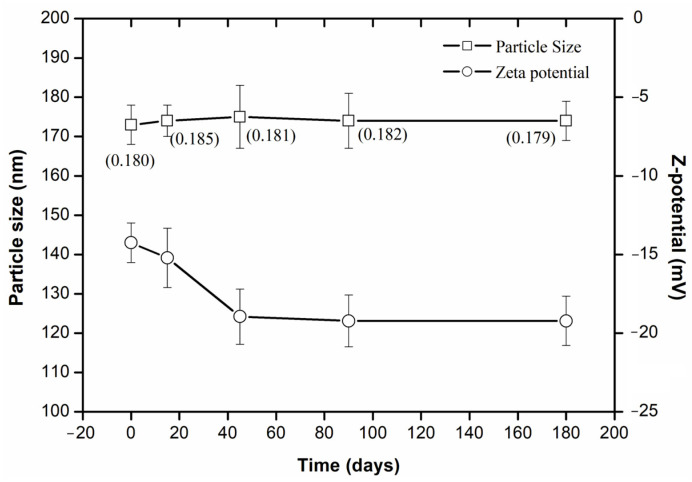
In shelf stability of the optimized bocaiuva oil-loaded nanoemulsion. In parenthesis is the polydispersity index.

The particle size remained constant for 180 days (Figure 5). However, the ζ-potential decreased during the first 45 days and then remained constant up to day 180, indicating nanoemulsion stabilization. This stabilization is due to an increase in the repulsive forces among the charged particles, which prevents aggregation and enhances stability. By better understanding these repulsive forces, we can significantly improve the stability and shelf life of the nanoemulsion, ensuring its efficacy and reliability in diverse applications.

**Figure 6:**
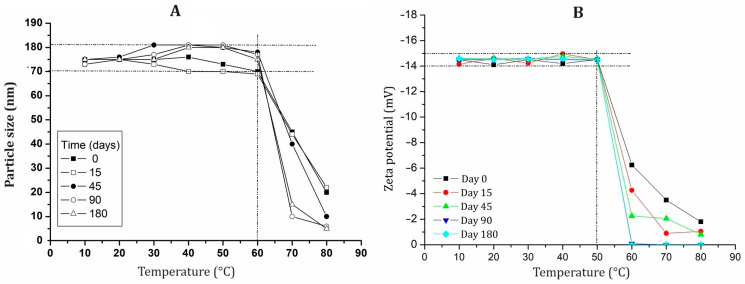
Effect of the temperature on the particle size (**A**) and ζ-potential (**B**) of the optimized bocaiuva fruit pulp oil-loaded nanoemulsion for 180 days.

The nanoemulsion was subjected to temperatures ranging from 10 to 80 °C to assess the combined effects of time and temperature on particle size (Figure 6A) and zeta potential (Figure 6B). From 0 to 60 °C, the particle size remained stable, ranging from 171 to 181 nm. However, beyond 60 °C, the size decreased to 120 nm across all time points. The zeta potential remained stable over time up to 50 °C; beyond this temperature, it increased to −6 mV at 60 °C on day 0 and eventually reached zero on day 180, indicating a loss of charge. Temperatures above 50 °C disrupt nanomicelles, leading to surfactant dehydration or micelle compression, resulting in micelle shrinkage [24].

These results are crucial for the preparation, storage, and usage of nanoemulsions. Understanding how temperature affects particle size and zeta potential helps in formulating nanoemulsions that maintain stability under varying conditions. This knowledge ensures that the nanoemulsion retains its intended properties, providing effective therapeutic delivery and accurate dosing throughout its shelf life. By optimizing storage conditions and usage parameters, we can enhance the overall efficacy and reliability of nanoemulsions in practical applications.

**Figure 7:**
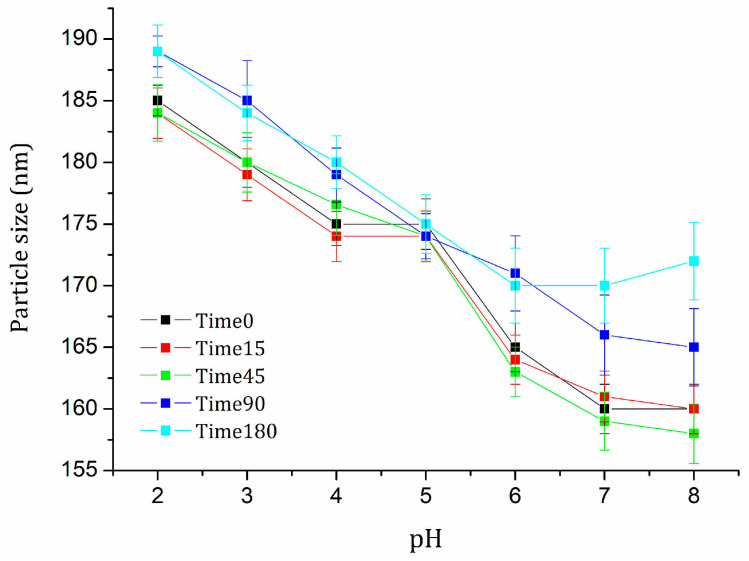
Effect of pH on the particle size of the nanoemulsion for 180 days.

The pH significantly impacts the stability of nanoemulsions. Different behaviors arise due to surfactant ionization and particle interactions at pH levels ranging from 2 to 8. Determining the pH of maximum stability is, therefore, essential for formulation processes using nanoemulsions as an active ingredient. The particle size of the nanoemulsion consistently decreased as the pH increased (Figure 7). This may have occurred because Tween 80 and Span 80 in acidic solutions are protonated, leading to increased positive charges around the particles, which in turn increases their size. Notably, at pH 5, the particle size remained statistically constant over time (F-test 1.25, *p*-value = 0.2415).

In addition to understanding the impact of temperature, it is crucial to comprehend how pH affects the properties of nanoemulsions. This knowledge is vital for the preparation, storage, and usage of bocaiuva oil-loaded nanoemulsions at all scales. By identifying the optimal pH for stability, formulations can be developed to maintain consistent particle sizes and, in consequence, consistent physicochemical properties. This ensures the nanoemulsion’s effectiveness and reliability in therapeutic delivery and other applications. Proper pH adjustments during preparation can enhance the stability and longevity of nanoemulsions, making them suitable for various storage conditions and ensuring precise dosing during use.

The physical and chemical on-shelf stability studies of the nanoemulsion underscore the success of its formulation and optimization. This preliminary investigation suggests that to maintain stability for at least 180 days, the optimized nanoemulsion should be stored at temperatures below 50 °C and kept at a pH between 4 and 5. Under these conditions, the nanoemulsion remains stable and effective. By following these guidelines, we can maximize the nanoemulsion’s performance and ensure consistent results in biological and other practical applications. Consequently, the optimized and stable nanoemulsion was used for biological assays.

### 2.5. Alpha-Glycosidase and Pancreatic Lipase Inhibitory Effect

Alpha-glucosidase inhibitors constitute a critical therapeutic class in the management of diabetes mellitus. The nanoemulsion shows a potent inhibition of α-glucosidase (IC_50_ of 43.21 µg/mL, Table 4). In contrast, the blank nanoemulsion did not inhibit it. The nanoemulsion’s inhibitory potency surpassed that of acarbose (IC_50_ of 63.08 µg/mL) and bocaiuva oil (IC_50_ > 250 µg/mL). The nanosized droplets loaded with fatty acids, carotenoids, and phenolic compounds create a large effective surface, enhancing their interaction with α-glucosidase, thus improving the effect. These results suggest that nanoemulsions can effectively be used for diabetes management; however, further research is necessary to validate this statement.

Pancreatic lipase plays a crucial role in lipid digestion, which is beneficial for patients with hyperlipidemia and/or cardiovascular diseases [25]. However, there are few pancreatic lipase inhibitors available on the market [26]. Lipase inhibitors do not selectively block the absorption of a particular type of fat; instead, they impact the overall fat absorption [27]. The nanoemulsion produced a strong inhibition of pancreatic lipase (IC_50_ 41.99 µg/mL, Table 4). On the contrary, the blank nanoemulsion did not inhibit it, and bocaiuva oil showed a weak inhibitory effect (IC_50_ > 250 µg/mL). The effect of the nanoemulsion was weaker than that of orlistat (IC_50_ 0.24 µg/mL). The inhibitory effect of the nanoemulsion was 10-fold greater than the effect of bocaiuva oil.

Micelles in the nanoemulsion loaded with bocaiuva oil include carotenoids, phenolics, oleic, linoleic, and palmitic acids. Carotenoids inhibit pancreatic lipase through free radical scavenging mechanisms [28,29]. Polyphenols interact with enzymes through various mechanisms, including hydrophobic interactions, hydrogen bonding, and ionic bonding, modifying the structure and function of pancreatic lipase, leading to inhibition [28]. Additionally, oleic and linoleic acids also inhibit pancreatic lipase [30]. All these compounds work synergistically across the high surface area of the nanoemulsion, contributing to the nanoemulsion’s potent inhibitory effect. The nanoemulsion could play a key role in controlling postprandial hyperglycemia and hyperlipidemia in diabetics, obese people, and patients with cardiovascular diseases [31].

### 2.6. Antiglycant Effect

Glycation is a chemical reaction where sugar molecules bind to proteins or fats, leading to the formation of harmful compounds called advanced glycation end products (AGEs). AGEs accumulate in tissues, accelerating the progression of diabetes, cardiovascular disease, and neurodegenerative disorders [32]. Antiglycant drugs prevent or delay the onset of diabetic complications [33]. The nanoemulsion’s antiglycant effect (IC_50_ 42.08 μg/mL, Table 5) was more potent than that of quercetin (IC_50_ 75.36 μg/mL) and aminoguanidine (IC_50_ 74.50 μg/mL). The blank nanoemulsion did not show antiglycant effects. The antiglycation mechanism of the nanoemulsion appears to be multifaceted due to its fatty acid composition, phenolics, and carotenes. The antioxidant effects of oleic acid and carotenes produce free radical scavenging, minimizing damage to living cells [33]. Polyphenol protects cells from damage induced by glycation processes, contributing to the antiglycation effect [32,33]. On the other hand, the large surface area of the nanoemulsion enhances the interaction of the nanoemulsion components with molecules and tissues. All these factors act synergistically to provide the nanoemulsion’s strong antiglycation effect. Thus, the nanoemulsion emerges as a promising nanomaterial that could be used in diabetics, obese people, and those with cardiovascular diseases. Nonetheless, further research is needed to elucidate the real utility of the nanoemulsion in these health conditions.

### 2.7. Cytotoxic Effect

The discovery of novel therapeutic alternatives that are less invasive and have fewer adverse effects in oncologic patients is a global imperative. Currently, many drugs used for cancer treatment exhibit high toxicity toward normal cells, a phenomenon known as low selectivity. The selectivity index, which measures a drug’s preferential action against cancer cells compared to healthy cells, is crucial for effective therapy. A selectivity index greater than three indicates a favorable therapeutic window, which is essential for the development of anticancer drugs [34]. Substances with a selectivity index greater than three are considered promising for targeting cancer cells while sparing normal tissues. 

Table 6 shows the cytotoxic effect of the nanoemulsion against five cancer cell lines after 48 h of treatment. The blank nanoemulsion (a nanoemulsion prepared in the same way but without bocaiuva oil) exhibited no cytotoxicity against cancer cells, with an inhibition percentage of less than 2%. In contrast, bocaiuva oil alone showed cytotoxicity specifically against prostate cancer cells (IC_50_ 66.25 μg/mL), with a favorable selectivity index (3.77). The nanoemulsion exhibited IC_50_ values > 250 μg/mL against normal cells (HFF1 and NIH/3T3). In both cases, the cytotoxicity was significantly lower and statistically different from that produced by doxorubicin (for HFF1: *t*-test = 40.57, *p* = 0.0002; for NIH/3T3: *t*-test = 3.36, *p* = 0.0280). The nanoemulsion showed promising cytotoxicity and selectivity against breast (MDA-MB 231 and MCF-7), renal (786-0) and prostate (PC-03) cancer cells.

While the nanoemulsion demonstrated excellent anti-proliferative effects against various cancer cell lines, bocaiuva oil showed specific anti-proliferative activity against prostate cancer cells. The cytotoxic effect of the nanoemulsion was three times greater against prostate cancer cells compared to bocaiuva oil and displayed a higher selectivity index (Table 6), probably due to the higher surface area offered by the nanosize. Nonetheless, these results suggest that, if appropriately formulated (e.g., in soft gelatin capsules), bocaiuva oil could serve as a viable therapeutic option for prostate cancer. This approach represents a promising strategy for harnessing this natural product in its native form.

The high content of oleic acid, polyphenols, and carotenoids in the nanoemulsion significantly contributes to its potent cytotoxic effects. The predominance of oleic acid, as the main component of the oil, plays a crucial role in this activity. Oleic acid can induce apoptosis in cancer cells by activating caspase enzymes and increasing pro-apoptotic proteins [35]. Additionally, oleic acid can inhibit cell proliferation by arresting the cell cycle and downregulating growth factor signaling pathways [36]. The combined effects of oleic acid with other fatty acids, such as palmitic acid and stearic acid, may further enhance the nanoemulsion’s cytotoxicity.

The presence of polyphenols and carotenoids in the nanoemulsion contributes to its selectivity towards cancer cells [37]. Polyphenols possess strong antioxidant properties and have been shown to enhance the efficacy of other anticancer agents when encapsulated in nanoformulations, improving their bioavailability and therapeutic potential [10]. They can inhibit cell growth and induce apoptosis through multiple pathways, including the modulation of signaling pathways associated with cell survival and proliferation [10].

Carotenoids present in bocaiuva oil can enhance the cytotoxic effects of nanoemulsions against cancer cells. These carotenoids can induce apoptosis, inhibit cell cycle progression, and prevent metastasis by influencing oncogenic and tumor suppressor proteins [38]. Additionally, the high surface area of the nanoemulsion boosts interactions with neoplastic cells, thereby amplifying its cytotoxic potential [39]. The high surface area produced by the nanoemulsion, combined with the synergistic action of phenolics, carotenoids, and fatty acids, may result in more selective and effective cytotoxicity against cancer cells while minimizing damage to normal cells.

The nanoemulsion exhibits varying degrees of selectivity against different cancer cell lines, as indicated by their selectivity indices. The varying IC_50_ values for different cancer cell lines highlight the nanoemulsion’s effectiveness and selectivity. Different cell lines show varying sensitivities to the nanoemulsion, with IC_50_ values ranging from 19.13 μg/mL (PC-03) to 212.32 μg/mL (HepG2). This variation suggests that PC-03 cells are more susceptible due to intrinsic factors like mutations and metabolic pathways [39]. Lower IC_50_ values indicate higher potency, beneficial for targeting specific cancers while minimizing toxicity to normal cells.

The highest selectivity index was observed against PC-03 prostate cancer cells, with 13.07, followed by MCF-7 (9.26) and MDA-MB 231 (breast cancer cells, with 9.18), and renal cancer cells 786-0 (3.04). However, the nanoemulsion showed low selectivity against liver cancer cells (HepG2), with a selectivity index of 1.18, indicating a narrower therapeutic window and potential toxicity concerns for these cancer types. Selectivity index values greater than three for the nanoemulsion against breast, prostate, and renal cancer cells strongly suggest its potential as an effective therapeutic agent with minimal off-target toxicity. This profile indicates a reduced likelihood of adverse effects during post-chemotherapy treatment compared to conventional chemotherapeutics [39,40]. These findings suggest that the nanoemulsion could be highly effective in treating prostate and breast cancers, and effective against renal cancer, all with a favorable therapeutic window.

It is important to note that the cytotoxicity and selectivity data presented here are based on in vitro studies using cell lines. The successful translation of these findings to in vivo and clinical settings may depend on addressing factors such as pharmacokinetics, biodistribution, and the tumor microenvironment. Therefore, further studies are needed to evaluate the nanoemulsion’s efficacy in animal models and to explore its potential for clinical applications.

It is notable that, despite the fruit pulp oil of *Acrocomia aculeata* (Bocaiuva) palm tree not having a unique composition, it presented a broad spectrum of important activities. At the nanoscale, the properties of materials can change significantly due to several factors. Firstly, they can change due to the increased surface area. At the nanoscale, the surface-area-to-volume ratio of materials increases dramatically, allowing for more interactions with the surrounding environment, leading to enhanced chemical reactivity and catalytic activity. On the other hand, at the nanoscale, quantum mechanical effects become significant, modifying the electronic, optical, and magnetic properties of materials, often resulting in novel behaviors. On the other side, numerous materials at the nanoscale show size-dependent and shape-dependent properties, for example, the membrane permeability of carbon nanotubes. Nanoparticulate materials change surface energy. A significant portion of the atoms and molecules present on the surface increase their surface energy. This can result in changes in melting points, phase transitions, and the solubility of the materials, and this makes all the difference in nanoformulated pharmaceuticals. These factors contribute to the enhanced activity and novel behaviors of materials at the nanoscale compared to their pristine counterparts. Understanding these changes is crucial for the development of nanotechnology applications in various fields such as medicine, electronics, and energy.

### 2.8. Hemolytic Effect and Platelet Aggregation

Preclinical toxicological evaluation of nanoparticles is crucial in the development of new drugs to assess potential adverse effects and risks to humans from exposure to nanoparticles. Two essential toxicological tests for evaluating the impact of nanoparticles on human health are the hemolysis effect and the platelet aggregation test.

**Figure 8:**
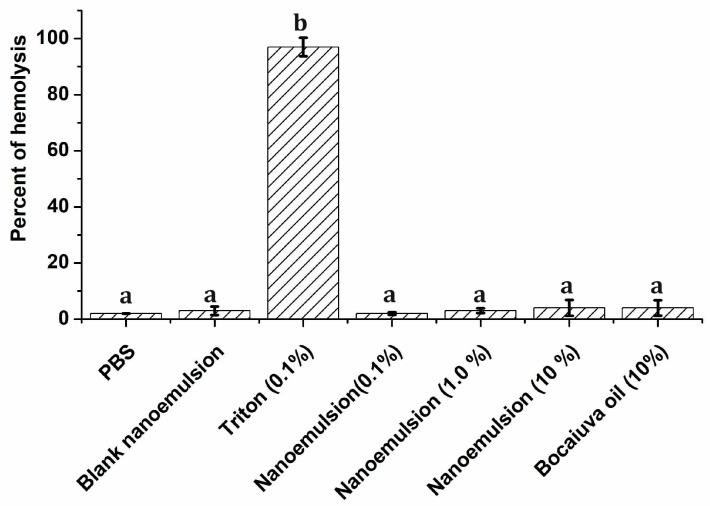
Hemolytic effect of the bocaiuva oil and bocaiuva oil-loaded nanoemulsion on red blood cells. Different letters indicate statistically significant differences at *p* < 0.05.

Hemolysis refers to the rupture of red blood cells and the release of their contents into the blood. Hemolysis testing is a key step in determining the biocompatibility of nanoparticles [41]. In this test, it was observed that both PBS and the blank nanoemulsion produced a hemolytic effect of 1.50% that was not statistically different from the effect of the nanoemulsion (at all concentrations) and bocaiuva oil at 10% (Figure 8). Triton 0.1% produced 99% hemolysis, and its effect was statistically different from all the other groups. The protective effect of bocaiuva oil and the nanoemulsion on erythrocytes could be related to both the bocaiuva oil composition and the nanoemulsion’s physicochemical properties. Bocaiuva oil contains fatty acids, carotenoids, and phenolic compounds, which are associated with cell membrane protection; consequently, they are less likely to disrupt the erythrocyte cell membrane [41]. Thus, the combination of biocompatible antioxidant compounds and the stable delivery system contribute to the non-hemolytic nature of both bocaiuva oil and the nanoemulsion.

**Figure 9:**
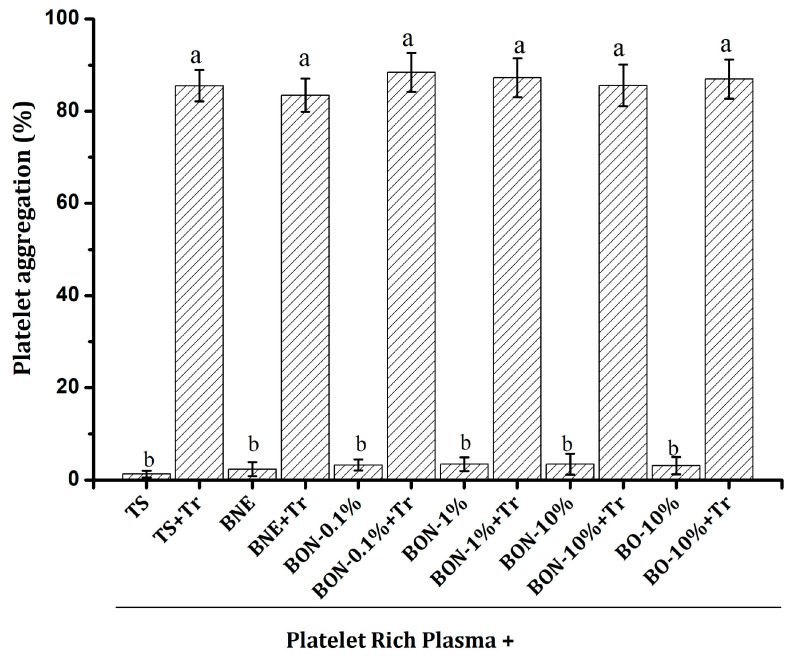
Effect of the bocaiuva oil and bocaiuva oil-loaded nanoemulsion on platelet aggregation. TS, Tyrode’s solution; Tr, thrombin solution; BNE, blank nanoemulsion; BON, bocaiuva oil nanoemulsion; BO, bocaiuva oil. Different letters indicate statistically significant differences at *p* < 0.05.

Platelet aggregation is the process by which platelets stick together at sites of vascular injury. Nanoparticles can either promote or inhibit platelet aggregation [42]. This process is important for effective hemostasis after platelets first adhere to an injury site. Platelet aggregation is also critical for thrombosis and hemostatic plug formation [43]. Assessing the effect of nanoparticles on human platelets in vitro is essential for quickly screening their potential anticoagulant or thrombogenic properties [42]. In our study, neither the bocaiuva oil nor the nanoemulsion at any concentration showed signs of platelet aggregation (Figure 9). On the other hand, none of the substances assessed, including positive and negative controls, prevented thrombin-induced aggregation. In all cases, the platelet aggregation effect of thrombin was higher and statistically different from the same solution without thrombin. Neither the bocaiuva oil nor the nanoemulsion exhibited anticoagulant or thrombogenic effects. The nanoemulsion’s kinetic stability ensures that it does not readily release its contents, preventing abrupt interactions with platelets that could compromise their integrity and function.

### 2.9. Future Perspectives

Although the nanoemulsion loaded with bocaiuva oil exhibits less activity (higher IC_50_) against cancer cells than doxorubicin, it demonstrates significant antiproliferative properties. Furthermore, the nanoemulsion holds promise as a cancer treatment due to its potentially reduced or absent adverse effects and numerous benefits stemming from its rich composition of essential fatty acids, phenols, and carotenoids. Unlike doxorubicin, which possesses high activity but low selectivity, resulting in numerous side effects for patients, the bocaiuva oil-loaded nanoemulsion represents a promising new therapeutic alternative for cancer treatment.

Future strategies to enhance the nanoemulsion of bocaiuva oil, particularly in terms of resistance to enzymatic degradation, physiological pH fluctuations, and temperature variations in biological environments, could involve the incorporation of advanced surfactants and co-surfactants that stabilize the emulsion at varying pH levels and temperatures. On the other hand, employing high-pressure homogenization techniques to achieve smaller droplet sizes could enhance the stability and bioavailability of the nanoemulsion. These approaches collectively aim to optimize the delivery and efficacy of bocaiuva oil in various biological environments. At this moment, the results obtained here support other studies seeking to improve the stability, absorption, and permeation into animal membranes in preclinical studies.

## 3. Materials and Methods

### 3.1. Plant Material

The fruits of *A. aculeata* were collected from bocaiuva palm trees that grew naturally, forming a small homestead in the Federal University of Mato Grosso do Sul, in Campo Grande, Mato Grosso do Sul, Brazil (−20°.50′00.1″ S, −54°.36′45.7″ W). The Department of Botany, Federal University of Mato Grosso do Sul confirmed the species’ identification under the registration code CGMS-4477. The fruit pulp was separated from the kernel and immediately used for oil extraction.

### 3.2. Oil Extraction

Bocaiuva oil was extracted at room temperature, using a bench double-plate electric press (Lith, Wuhan, China). One kilogram of the pulp was pressed at 500 kN, keeping the mass under pressure for 15 min, to obtain a crude pressing oil. Posteriorly, the crude oil was filtered using a 100 mL vacuum cartridge of diatomaceous earth sorbent (celite, Thelos, Redland Bay, Australia) for rapid sample preparation to obtain a limpid bocaiuva oil. The yield of the process was 25.50% (mass of oil per kg of the dried material). The oil was kept in an amber flask until used.

### 3.3. Organoleptic and Physicochemical Characterization

The organoleptic properties of bocaiuva oil (color, aroma, and appearance) were assessed based on perception. The refractive index [44], relative density, iodine index, peroxide index, saponification value, and acidity value were evaluated [45].

### 3.4. Phenolic Content

The Folin–Ciocalteu spectrophotometric method was employed [46]. The amount of 3 mL of bocaiuva oil was mixed with 75% ethanol solution (10 mL). The mixture was mechanically stirred for 2 h and left in the dark for 24 h. Subsequently, the liquid was centrifuged at 5000 rpm (LKP, São Paulo, Brazil). Aliquots of 1 mL of the ethanolic phase were used. A calibration curve was constructed using gallic acid as the standard (Sigma, Cream Ridge, NJ, USA). The results were expressed as the gallic acid equivalent of three replications.

### 3.5. Carotenoids as β-Carotene

This was evaluated spectrophotometrically (Shimadzu, Japan) as per Rodríguez-Amaya [47]. In a volumetric flask (100 mL), 3 mL of bocaiuva oil was dissolved in n-hexano and the final volume was complete. The oil solution was filtrated by a Millipore membrane (0.45 µm) for eliminating possible mechanical impurities. Absorbance measurements were conducted using n-hexane as a blank. The molar extinction coefficient of *β*-carotene in n-hexane at 453 nm (2592 mol^−1^cm^−1^) was used. The carotenoid content (TC, Equation (1)) was expressed as β-carotene using Equation (2):

(2) $$TC\;(µg/100\;g)=\frac{A*V*10000}{\varepsilon *m}$$

where A is the absorbance of the sample; V the sample volume; ε the molar absorptivity of β-carotene, and m the mass of the sample. The results were expressed as the mean ± standard deviation of three replications.

### 3.6. GC/MS Analysis

A derivatization process of the oil fatty acids was conducted. GC-MS analysis was performed using a Mega 2 series gas chromatograph coupled to a Shimadzu GCMS-QP500 mass spectrometer (GC/MS) (Tokyo, Japan). A 30 m × 0.32 mm capillary column with a 0.25 mm thick layer (66DB-5MS, Agilent Technologies, Santa Clara, CA, USA) was used as the stationary phase. Helium was used as the carrier gas at a flow rate of 1 mL/min, using a split ratio of 1:10. The injector temperature was set at 250 °C. The oven temperature was scheduled to start at 130 °C for 10 min and then increased to 250 °C at a rate of 5 °C/min, with a final temperature for 10 min. Mass spectra were acquired using a mass range of *m*/*z* 40–500, an interface temperature of 250 °C, and an ion source temperature of 220 °C. The solvent cutoff time was set to 3 min, and the event time was 0.20. The scan rate was set to 2500. The composition (in percent) was calculated using the peak normalization method.

### 3.7. Nanoemulsion Development

**Figure 10:**
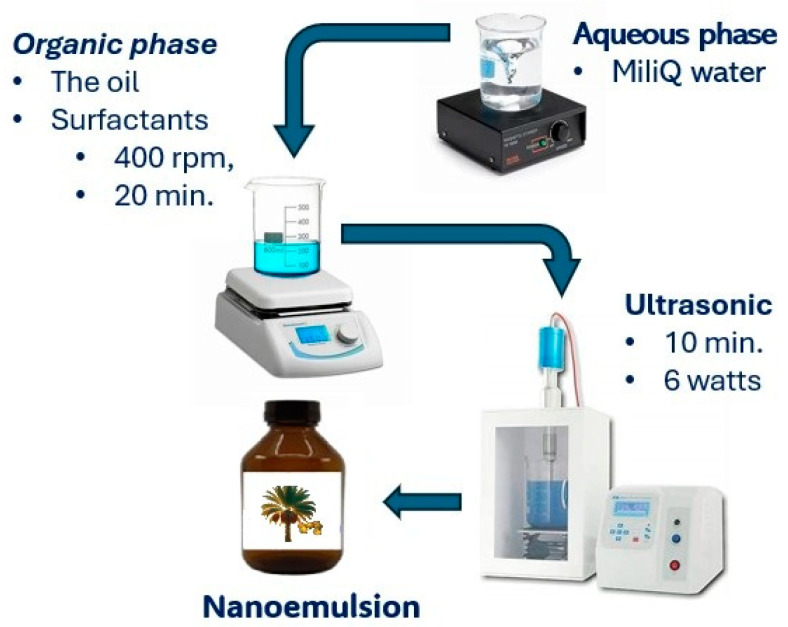
Nanoemulsion preparation process.

The nanoemulsion was formulated using the phase inversion methodology, as described by [15]. The organic phase, consisting of bocaiuva oil (5%) and surfactants (5%, mixtures of Sorbitan Monooleate (Span 80): Polysorbate 80 (Tween 80), was stirred at 400 rpm (Fisaton, Brazil) at 25 °C for 20 min. The aqueous phase, composed of 90% of Milli-Q water, was added to the organic phase (1 mL/min) under continuous stirring at 400 rpm (Fisaton, Sa Paulo, Brazil). The stirring continued for an additional 20 min after the addition of the aqueous phase. The nanoemulsion was finally sonicated (10 min, 6 watts) to reduce and stabilize the particle size [3]. Finally, the initial volume of the nanoemulsion (50 mL) was restored with Milli-Q water (Figure 10).

### 3.8. Required Hydrophilic–Lipophilic Balance

The required Hydrophilic–Lipophilic Balance (HLBr) of bocaiuva oil was determined. A set of nanoemulsions were prepared using HLB values from 4.3 to 15, obtained by the mixture of different proportions of Span 80 (HLB 4.3) and Tween 80 (HLB 15). The temperature was kept at 25 ± 1 °C. The surfactant HLB that produced the most stable nanoemulsion with the smallest particle size was set as the bocaiuva oil’s HLBr [15].

### 3.9. Particle Size, ζ-Potential, and pH

Particle size and the polydispersity index were measured by Photon Correlation Spectroscopy (PCS) using a Zetasizer Nano-ZS instrument (Malvern, Worcestershire, UK). The ζ-potential was determined by Electrophoretic Light Scattering (ELS) using a Zetasizer Nano-ZS instrument (Malvern, Worcestershire, UK). All measurements were performed in triplicate, and the results were reported as the mean ± standard deviation [3].

### 3.10. Scanning Electronic Microscopy

Scanning Electronic Microscopy (SEM) was used to evaluate the size and morphology of the nanoparticles. The liquid nanoemulsion (1 mL) was dispersed in 2 mL of absolute ethanol using a micropipette and immediately deposited on the carbon grid for the image acquisition. A CM20 high-voltage scanning electronic microscope (Phillips, Amsterdam, The Netherlands) was used, with a LaB6 electron source, a voltage of 20 kV, and 15k of augment. The Digital Micrograph^®^ 3.5 software from Gatan Microscopy Suite^®^ (GMS) was used to automatically determine the size and the size distribution of nanoparticles in the nanoemulsion.

### 3.11. pH Evaluation

The pH was evaluated using a pH-meter (Tecnopon, São Paulo, Brazil). The equipment was calibrated using buffer solutions (Alphatec, São Paulo, Brazil) at pH 4, 7, and 10. Two milliliters of the nanoemulsion was diluted in Milli-Q water (4 mL). The assay was made in triplicate, and the results were expressed as the mean ± standard deviation [3]. The measures were made 24 and 72 h after the preparation. The value was reported at 72 h.

### 3.12. Nanoemulsion Optimization

The process parameters selected as factors for nanoemulsion optimization are based on a previous screening in which it was observed that potencies below 5 watts for less than 5 min resulted in phase separation and creaming, preventing nanoemulsion formation. In the same way, ultrasonic potency exceeding nine watts, independently of the sonication time, led to increased temperature and subsequent phase separation. Thus, for the optimization, an ultrasonic potency of 5–8 watts (low and higher level, respectively) and sonication time of 5 and 15 min were selected. The optimization was performed using a D-optimal Response Surface design [22,48]. The particle size and polydispersity index were measured as responses 24 and 72 h after the nanoemulsion preparation. Design Expert 6.0 (StatEase, Minneapolis, MN, USA) was used for data analysis. The statistically significant level was set at 0.05.

### 3.13. On-Shelf Physical and Chemical Stability

The selected nanoemulsion was transferred into an amber bottle and stored at 25 ± 1 °C. The particle size, polydispersity index, and ζ-potential were measured at 0, 15, 45, 90, and 180 days, using the procedures described in 2.9. The bocaiuva oil content in the optimized nanoemulsion was monitored at time 0 (the exact amount of bocaiuva oil used for nanoemulsion preparation), 90, and 180 days. The assay was carried out using Gas Chromatography, using the same technique described in 2.6. At each time, the percentage of oil contained in the nanoemulsion was calculated. The mean ± standard deviation of three replicates was reported.

The effect of temperature (between 10 and 70 °C) on the particle size, polydispersity index, and ζ-potential was evaluated at 10, 20, 30, 40, 50, 60, and 70 °C. The assay was performed in triplicate, and the results were expressed (graphically) as the mean ± standard deviation.

The effect of the pH (between 2 and 8 pH-units) on nanoemulsion particle size and ζ-potential was evaluated over time at days 0, 15, 45, 90, and 180. The nanoemulsion was stored for 180 days at 25 ± 1 °C. The assay was performed in an MTZ2 Automatic titrator (Malvern, UK) coupled to Zetasizer Nano ZS (Malvern, UK). An amount of 2 mL of nanoemulsion was diluted in 4 mL of Milli-Q water. After that, a disposable polycarbonate Z-cell cuvette was filled with the nanoemulsion dilution. The titration was carried out (automatically) by adding NaOH 0.1 molL^−1^ and HCl 0.1 molL^−1^ as titrating solutions to reach the desired pH. The cuvette was allowed to rest for five minutes at each pH before the measurement. The assay was carried out in triplicate, and the results were expressed graphically as the mean ± standard deviation.

### 3.14. Alpha-Glucosidase Inhibition

An α-glucosidase activity assay kit (Sigma Aldrich, St. Louis, MO, USA), catalog number MAK123, was used. The α-glucosidase inhibition was evaluated by a reaction in which α-glucosidase hydrolyzes p-nitrophenyl-α-D-glucopyranoside. The reaction produces a colored product (detectable at 405 nm) in a concentration proportional to α-glucosidase activity. The assay was carried out according to the manufacturer’s instructions. Acarbose was used as the reference drug.

### 3.15. Pancreatic Lipase Inhibition

The assay was performed using a Lipase Assay Kit (catalogue number MAK482, Sigma Aldrich (St. Louis, MO, USA) according to manufacturer’s instructions. The assay is based on the dimercaptopropanol tributyrate (BALB) method, in which -SH groups formed from lipase cleavage of BALB react with 5,5′-dithiobis (2-nitrobenzoic acid) (DTNB), forming a colored product, which is measured at 412 nm, and is proportional to the enzyme activity. Orlistat was used as the standard inhibitory substance.

### 3.16. Antiglycant Activity—Oxidative Pathway

An amount of 10 mL of reaction mixture was prepared by mixing a phosphate buffer (0.20 M, pH 7.4) containing BSA (10 mg/mL), glyoxal (30 mM), and sodium azide (3 mM). The reaction mixture (300 μL) was incubated with the nanoemulsion (100 μL) at concentrations of 10, 25, 50, 75, and 100 μg/mL. Quercetin was used as the reference and DMSO was used as the negative control. The blank nanoemulsion (50 μg) dissolved in PBS (50 μL) was used as the blank. Microplates were incubated (37 °C, 72 h, in the dark). The fluorescence intensity was measured at 370 nm (excitation) and 447 nm (emission) using a spectrofluorometer RF-1500 (Shimadzu^®^, Tokyo, Japan) [48]. The percentage of inhibition was used for estimating the IC_50_. The software GraphPad Prism^®^ 6.0 was used. The percent of inhibition was calculated using Equation (3):

(3) $$\%\;I=100-\left[\frac{F_{a/p}}{F_{C}}\right]*100$$

where F_a/p_ is the blank fluorescence minus the sample fluorescence, and F_C_ is the blank fluorescence minus the control fluorescence. The results were expressed as the mean ± standard deviation of three replications.

### 3.17. Antiglycant Activity—Non-Oxidative Pathway

The assay was performed in the same conditions used for the oxidative pathway, but using fructose (0.1 mM) instead of glyoxal. Aminoguanidine was used as the antiglycant reference drug. The results were expressed as the mean ± standard deviation of three replications [48].

### 3.18. Antiproliferative Effect

In this assay, six cell lineages were used, namely, HepG2 (ATCC HB-8065), HFF-1 (ATCC SCRC-1041), MCF7 (ATCC HTB-22), MDA-MB-231 (ATCC HTB-26), and PC-3 (ATCC CRL-1435), obtained from the American Type Culture Collection (Sigma-Aldrich Products, Manassas, VA, USA). The NIH/3T3 (ATCC CRL-165) cells were obtained from the Rio de Janeiro Cell Bank (Duque de Caxia, Brazil) [3].

The HepG2, HFF-1, and NIH/3T3 cells were cultured in DMEM medium (Gibco-Invitrogen, Brazil). MCF7, MDA-MB-231, and PC-3 cells were cultured in RPMI 1640 medium (Gibco-Invitrogen, São Paulo, Brazil). The culture media were supplemented with 10% inactivated fetal bovine serum (Gibco-Invitrogen, São Paulo, Brazil). The cells were kept at 37 °C in a 5% CO_2_ atmosphere up to a cell density of 5 × 10^3^ cells/well. After 24 h of cell adaptation, they were treated (for 48 h) with the nanoemulsion at concentrations ranging from 0.25 to 250 µg/mL and 0.025 to 25 µg/mL of doxorubicin. DMSO was used as the control, and doxorubicin as the positive control; a blank nanoemulsion was used as the negative control. The optical density (540 nm) was measured in a Spectramax 190 microplate reader (Marshall scientific, Hampton, NH, USA). The percentage of growth inhibition was calculated. The results were expressed as the mean of three independent experiments (n = 3), and the IC_50_ was calculated using the GraphPad Prism^®^ 6.0 software.

#### Selectivity Index

This was calculated as the ratio between the IC_50_ of the nanoemulsion in non-tumoral cells NIH/3T3 and the IC_50_ of tumoral cells (Equation (4)) [34].

(4) $$Selectivity\;index=\frac{IC50\;(NIH/3T3)}{IC50\;(Neoplastic\;cells)}$$

### 3.19. Effect of Bocaiuva Oil and Nanoemulsion on Blood Cells

#### Hemolytic Effect

Blood samples were collected from twenty healthy volunteers aged 18–25 years, who had not consumed any medication for at least 14 days prior to the study. The research protocol was approved by the Ethics and Research Committee of the HEMOAM Foundation (Opinion No.: 4,982,395) (CAAE 51257921.2.0000.0009), 12 October 2021. The assay was performed in the Virology Laboratory, INPA—National Institute for Amazonian Research. A sample of heparinized whole blood (5 mL) diluted in 5 mL of PBS (pH 7.4) was centrifugated at 3.7 krpm for 5 min at 5 °C (Analytical Ultracentrifuge, Optima XPN 80, São Paulo, Brazil). Red blood cells were separated from the supernatant and resuspended in 5 mL of PBS. Aliquots of 200 µL of red blood suspension were incubated at 37 °C for one hour with 200 µL of PBS (control dilution), the blank nanoemulsion (negative control), 0.1% Triton solution (positive control), the nanoemulsion at 0.1, 1.0, and 10%, and bocaiuva oil at 10%. After the incubation, all samples were centrifuged at 3700 rpm for 5 min, and 100 µL of supernatant was used to read the absorbance at 595 nm [45]. The results were expressed as the mean ± standard deviation of three independent experiments (n = 3). The percentage of hemolysis was calculated by Equation (5):

(5) $$\%\;Hemolysis=\frac{As-Anc}{Apc-Anc}*\;100$$

where *As* is the absorbance of the sample; *Anc* is the absorbance of the negative control (blank nanoemulsion); and *Apc* is the absorbance of the positive control (Triton solution).

### 3.20. Platelet Aggregation Test

Platelet aggregation was assessed using the single cell counting method, using a Helena AggRAM semi-automated light transmission aggregometer (Northeast of England, UK). Blood samples were collected from twenty healthy volunteers aged 18–25 years, who had abstained from any medication known to influence platelet function for at least 14 days prior to the study. The research protocol was approved by the Ethics and Research Committee of the HEMOAM Foundation (Opinion No.: 4,982,395) (CAAE 51257921.2.0000.0009). The assay was performed in the Virology Laboratory, INPA—National Institute for Amazonian Research. Platelet-rich plasma (PRP, 805,500 platelets µL^−1^) was prepared by centrifuging the blood at 2500 rpm for 20 min. Subsequently, PRP was diluted with Tyrode’s solution to achieve a final concentration of 250,000 platelets µL^−1^, using the same equipment. Platelet-poor plasma (PPP) was prepared by further centrifuging the PRP at 15,000 rpm for 10 min at room temperature. Subsequently, 400 µL of PRP was mixed with 50 µL of test substances (blank nanoemulsion, nanoemulsion, and bocaiuva oil) and 50 µL of 0.02 UI/mL thrombin solution, which served as the platelet aggregation inducer. The samples were incubated at 37 °C for 5 min in an aggregometer (Chrono-log Corp., Havertown, PA, USA). The aggregation effect was evaluated in PRP with the addition of the test substance, both in the presence and absence of thrombin solution [40]. Data were expressed as a percentage of platelet aggregation, determined by transmittance values from the aggregometer, with PRP representing 100% aggregation and PPP representing 0%.

### 3.21. Statistical Analysis

One-way ANOVA followed by Tukey’s HSD test was performed to evaluate statistically significant differences among the experimental groups. A statistically significant difference was considered at *p* ≤ 0.05. StatGraphics^®^ Centurion XV.1 software (StatEase, Minneapolis, MN, USA) was used for the analysis.

## 4. Conclusions

In this study, the physicochemical properties of *Acrocomia aculeata* fruit pulp oil obtained by cold pressing were established as a starting point for standardizing the quality control parameters of the oil for subsequent production chains. A novel bocaiuva oil-loaded nanoemulsion was formulated and optimized, exhibiting a particle size of 173.6 nm and a zeta potential of −14.10 mV. The nanoemulsion efficiently inhibited α-glucosidase (IC_50_ 43.21 µg/mL) and pancreatic lipase (IC_50_ 41.99 µg/mL), and demonstrated a strong antiglycant effect through both oxidative (IC_50_ 18.36 µg/mL) and non-oxidative pathways (IC_50_ 16.33 µg/mL). The optimized nanoemulsion exhibited potent cytotoxicity, with good selectivity against prostate cancer (PC03, IC_50_ 19.13 µg/mL) and triple-negative breast cancer cells (MDA-MB 231, IC_50_ 27.22 µg/mL), without hemolytic effects, platelet aggregation, or anticoagulant effects. While the nanoemulsion emerges as a promising candidate for managing diabetes, hyperlipidemia, cardiovascular diseases, and certain types of cancer, further investigations are required to fully understand its benefits and mechanisms of action.

## Figures and Tables

**Table 1:** Physicochemical properties of the *Acrocomia aculeata* fruit pulp oil.

Characteristic	Value (n = 3)
Relative density (30 °C)	0.9000 ± 0.0001
Iodine index (g of I_2_/100 g)	74.50 ± 1.50
Refractive index (30 °C)	1.456 ± 0.001
Peroxide index (mEq/Kg)	4.50 ± 0.40
Saponification index (mg KOH/g)	133.00 ± 4.50
Acidity (mg/100 g)	0.92 ± 0.10
Total carotenoids (mg/100 g)	0.27 ± 0.01
Polyphenols (mg/100 g)	12.60 ± 0.30

**Table 2:** Fatty acid composition of the *Acrocomia aculeata* fruit pulp oil.

Fatty Acids	Chain	Rt	Ri	Content (%)
Saturated				
Hexanoic acid (caproic)	C6:0	5.15	974	0.22 ± 0.02
Octanoic acid (caprylic)	C8:0	7.26	1169	0.25 ± 0.02
Decanoic acid (capric)	C10:0	11.55	1365	0.13 ± 0.01
Dodecanoic acid (lauric)	C12:0	15.95	1548	0.85 ± 0.01
Tetradecanoic acid (myristic)	C14:0	21.49	1747	0.70 ± 0.01
Hexadecanoic acid (palmitic)	C16:0	27.86	1970	16.52 ± 0.15
Octadecanoic acid (stearic)	C18:0	33.28	2164	4.11 ± 0.15
Eicosanoic acid (arachidic)	C20:0	35.37	2369	0.20 ± 0.03
Docosanoic acid (behenic)	C22:0	41.58	2562	0.06 ± 0.03
Subtotal				23.04
Monounsaturated				
δ-9-cis-hexadecenoico acid (palmitoleic)	C16:1	25.36	1939	2.54 ± 0.01
cis-9-octadecenoic acid (oleic)	C18:1	30.75	2241	71.25 ± 2.21
Subtotal				73.79
Polyunsaturated				
cis-9,12-octadecadienoic acid (linoleic)	C18:2	32.46	2154	0.80 ± 0.04
cis-octadeca-9,12,15-trienoic acid (linolenic)	C18:3	31.05	2176	2.20 ± 0.33
Subtotal				3.00
Total fatty acids				99.83

Rt, retention time. Ri, literature retention index (from NIST chemistry webbook, SRD 69). (n = 3).

**Table 3:** Matrix outcome of the experimental design for nanoemulsion optimization.

Run	Factors		Responses			
	Potency	Time	24 h		72 h	
	(Watt)	(min.)	Size(nm)	PdI	Size (nm)	PdI
1	8	10	111.10 ± 0.56	0.245	374.50 ± 3.23	0.386
2	6	15	121.60 ± 1.27	0.235	-	-
3	7	15	98.31 ± 0.60	0.240	-	-
4	5	5	148.10 ± 0.21	0.252	428.70 ± 2.58	0.345
5	7	10	119.70 ± 1.24	0.244	327.10 ± 9.28	0.437
6	7	10	118.10 ± 0.21	0.251	200.40 ± 11.85	0.382
7	8	5	95.73 ± 1.25	0.249	-	-
8	6	5	145.40 ± 0.07	0.217	175.50 ± 21.47	0.308
9	6	15	108.70 ± 0.14	0.246	-	-
10	7	10	119.30 ± 0.77	0.248	-	-
11	6	10	125.80 ± 0.56	0.248	187.10 ± 8.27	0.348
12	7	5	136.80 ± 1.26	0.257	198.40 ± 8.47	0.357
13	7	10	121.3 ± 0.00	0.272	171.5 ± 2.58	0.472
14	8	15	136.40 ± 2.4	0.235	139.40 ± 1.75	0.207
15	7	15	103.30 ± 2.05	0.254	-	-

Sign (-) indicates phase separation at 72 h after the preparation. PdI, polydispersity index.

**Table 4:** Alpha-glucosidase and pancreatic lipase inhibitory effect of bocaiuva fruit pulp oil and nanoemulsion.

Tested Substances	α-Glucosidase IC_50_(µg/mL)	Pancreatic Lipase IC_50_ (µg/mL)
Blank nanoemulsion	Does not inhibit	Does not inhibit
Bocaiuva oil	>250	>250
Nanoemulsion	43.21 ± 2.15 ^a^	41.99 ± 3.48 ^a^
Acarbose^®^	63.08 ± 3.25 ^b^	Not tested
Orlistat^®^	Not tested	0.24 ± 0.04 ^b^

Different letters in a column denote statistically significant differences at *p* < 0.05.

**Table 5:** Antiglycant effect of the bocaiuva fruit pulp oil-loaded nanoemulsion.

Substances	Oxidative Pathway		Non-Oxidative Pathway	
	Inhibition (%) (at 100 μg/mL)	IC_50_ (μg/mL)	Inhibition (%) (at 100 μg/mL)	IC_50_ (μg/mL)
Blank nanoemulsion	No inhibition		No inhibition	Not tested
Nanoemulsion	42.08 ± 2.73 ^a^	18.36 ± 2.11 ^a^	38.29 ± 4.18 ^a^	16.33 ± 2.85 ^a^
Bocaiuva oil	92.41 ± 3.53 ^b^	57.33 ± 4.70 ^b^	>250	Not tested
Quercetin	75.36 ± 3.54 ^c^	42.37 ± 3.74 ^c^	Not tested	Not tested
Aminoguanidine	Not tested	Not tested	74.50 ± 3.37 ^b^	37.34 ± 1.71 ^b^
F-test, *p*-value	168.21, 0.0000	62.34, 0.0000	351.25, 0.0000	28.45, 0.0000

Different letters in the column indicate statistically significant differences at *p* < 0.05 from Tukey’s multiple range test.

**Table 6:** Antiproliferative effect of the *Acrocomia aculeata* fruit pulp oil-loaded nanoemulsion in carcinoma cell lines (n = 3).

Cell Line	Doxorubicin IC_50_ (μg/mL)	Bocaiuva Oil		Nanoemulsion	
		IC_50_ (μg/mL)	Selectivity Index	IC_50_ (μg/mL)	Selectivity Index
HFF1	2.45 ± 0.03 ^a^	>250	-	>250 ^b^	-
NIH/3T3	3.89 ± 0.41 ^a^	>250	-	>250 ^b^	-
MDA-MB 231	1.51 ± 0.03 ^a^	>250	-	27.22 ± 0.03 ^b^	9.18 ^b^
MCF-7	0.19 ± 0.01 ^a^	>250	-	26.99 ± 0.03 ^b^	9.26 ^b^
PC-03	0.28 ± 0.11 ^a^	66.25	3.77	19.13 ± 0.03 ^b^	13.07 ^a^
786-0	0.26 ± 0.12 ^a^	>250	-	82.29 ± 0.03 ^b^	3.04 ^c^
HepG2	0.25 ± 0.11 ^a^	>250	-	212.32 ± 0.03 ^b^	1.18 ^d^

HFF-1: normal fibroblast, derived from human foreskin; NIH/3T3: mouse embryonic fibroblasts; MDA-MB-231: a human triple-negative breast cancer cell line; MCF-7: a human breast cancer cell line; PC-03: a human non-small-cell lung cancer line; 786-0: human cell renal carcinoma; HepG2: a human hepatocellular carcinoma cell line. Different letters in a row indicate statistically significant differences at *p* < 0.05.

## Data Availability

Other data supporting this study can be obtained on demand. The authors declare that the data supporting the findings of this study are available from the corresponding authors upon reasonable request. Information that could compromise the privacy of the research participants is not available.
